# Supplementary material for: Time-series transcriptome analysis identified differentially expressed genes in broiler chicken infected with mixed Eimeria species
Source: Front Genet. 2022 Aug 8;13:886781. doi: 10.3389/fgene.2022.886781 (PMC9393255; doi:10.3389/fgene.2022.886781)
Supplement: Supplementary file 2 [file DataSheet1.ZIP › 7dpi_GO.Gsea.1625106495923/GOBP_PHOSPHATIDYLINOSITOL_3_PHOSPHATE_BIOSYNTHETIC_PROCESS.html]

Details for gene set GOBP\_PHOSPHATIDYLINOSITOL\_3\_PHOSPHATE\_BIOSYNTHETIC\_PROCESS[GSEA]

|  || Dataset | TMM\_7dpi\_gct\_format\_7dpi\_gct\_format.Class\_4dpi.cls #PC\_versus\_NC.Class\_4dpi.cls #PC\_versus\_NC\_repos |
| Phenotype | Class\_4dpi.cls#PC\_versus\_NC\_repos |
| Upregulated in class | 0 |
| GeneSet | GOBP\_PHOSPHATIDYLINOSITOL\_3\_PHOSPHATE\_BIOSYNTHETIC\_PROCESS |
| Enrichment Score (ES) | -0.73793113 |
| Normalized Enrichment Score (NES) | -1.8682595 |
| Nominal p-value | 5.2534806E-4 |
| FDR q-value | 0.08473579 |
| FWER p-Value | 0.6292 |
Table: GSEA Results Summary

  

Fig 1: Enrichment plot: GOBP\_PHOSPHATIDYLINOSITOL\_3\_PHOSPHATE\_BIOSYNTHETIC\_PROCESS      
 Profile of the Running ES Score & Positions of GeneSet Members on the Rank Ordered List

  

| SYMBOL | TITLE | RANK IN GENE LIST | RANK METRIC SCORE | RUNNING ES | CORE ENRICHMENT || 1 | PIK3C2B | na | 743 | 0.616 | -0.0096 | No |
| 2 | FIG4 | na | 5543 | -0.153 | -0.3967 | No |
| 3 | PIK3CG | na | 8478 | -0.481 | -0.6005 | No |
| 4 | PIK3C2G | na | 10128 | -0.737 | -0.6754 | Yes |
| 5 | PIK3CB | na | 10335 | -0.777 | -0.6267 | Yes |
| 6 | ATM | na | 10345 | -0.779 | -0.5614 | Yes |
| 7 | PIK3CD | na | 10707 | -0.855 | -0.5189 | Yes |
| 8 | PIK3CA | na | 10920 | -0.912 | -0.4592 | Yes |
| 9 | PIK3R3 | na | 11045 | -0.949 | -0.3890 | Yes |
| 10 | PIK3C3 | na | 11107 | -0.966 | -0.3121 | Yes |
| 11 | INPP4A | na | 11216 | -1.005 | -0.2359 | Yes |
| 12 | PIK3C2A | na | 11243 | -1.015 | -0.1519 | Yes |
| 13 | PIKFYVE | na | 11699 | -1.238 | -0.0848 | Yes |
| 14 | INPP4B | na | 11768 | -1.304 | 0.0202 | Yes |
Table: GSEA details [plain text format]

  

Fig 2: GOBP\_PHOSPHATIDYLINOSITOL\_3\_PHOSPHATE\_BIOSYNTHETIC\_PROCESS      
 Blue-Pink O' Gram in the Space of the Analyzed GeneSet

  

Fig 3: GOBP\_PHOSPHATIDYLINOSITOL\_3\_PHOSPHATE\_BIOSYNTHETIC\_PROCESS: Random ES distribution      
 Gene set null distribution of ES for **GOBP\_PHOSPHATIDYLINOSITOL\_3\_PHOSPHATE\_BIOSYNTHETIC\_PROCESS**
